# Effectiveness of the Smartphone Application mymobility® for Returning to Sports Activity After Medial Opening Wedge High Tibial Osteotomy: A Case Report

**DOI:** 10.7759/cureus.102529

**Published:** 2026-01-29

**Authors:** Toshiki Azuma, Kenichi Goshima, Kayo Oari, Ryosuke Hasama, Kentaro Sasaki

**Affiliations:** 1 Department of Rehabilitation Medicine, Kanazawa Munehiro Hospital, Kanazawa, JPN; 2 Department of Rehabilitation Medicine, Kinjo University, Hakusan, JPN; 3 Department of Orthopaedic Surgery, Kanazawa Munehiro Hospital, Kanazawa, JPN; 4 Department of Physical Medicine and Rehabilitation, Kinjo University, Hakusan, JPN

**Keywords:** 10 high tibial osteotomy, medial compartmental osteoarthritis, mymobility, return to sports, smartphone application

## Abstract

Medial opening wedge high tibial osteotomy (MOWHTO) is an effective surgical procedure for joint preservation in active patients with medial knee osteoarthritis (KOA). However, older patients and those with prolonged preoperative sports cessation often struggle to resume high-impact activities, such as running, after MOWHTO. Remote rehabilitation tools may help overcome these challenges. We report the case of an older woman with prolonged cessation of sports activity who resumed sports activity after MOWHTO with the help of the mymobility® smartphone application. A 71-year-old woman with Kellgren-Lawrence grade 3 medial KOA and -5° varus alignment underwent MOWHTO. She discontinued daily 7 km runs for two years owing to knee pain. The preoperative Knee Injury and Osteoarthritis Outcome Score (KOOS) scores were 53 for Symptoms, 50 for Pain, 61 for Activities of Daily Living, 25 for Sport and Recreation Function, and 6.25 for Quality of Life, and the Oxford Knee Score (OKS) was 31. Bone union was achieved on postoperative day (POD) 90. However, she maintained a low step count (approximately 2000 steps/day) and was unable to run owing to anxiety. We used mymobility® to provide chat-based guidance, step-count goals, and video assessments of jogging and running form. Activity progressively increased, and she achieved jogging ability on POD 150 and pain-free running ability on POD 180. Her Visual Analog Scale score improved to 0 mm, KOOS and OKS scores improved substantially, and step count increased to approximately 8000 steps/day. This case indicates that mymobility® can facilitate safe and timely return to sports after MOWHTO by improving motivation, providing remote supervision, and enabling individualized activity progression. mymobility® may be particularly effective for older patients or those who cannot access specialized postoperative rehabilitation services.

## Introduction

Medial opening wedge high tibial osteotomy (MOWHTO) is a widely used surgical procedure for joint preservation in active patients with medial compartment knee osteoarthritis (KOA). MOWHTO yields favorable outcomes and enables many patients to return to work or sports [1,2]. The return-to-sport (RTS) rate has been reported to range from 55% to 100%, with most patients returning to work or sports between six and 14 months postoperatively [2,3]. However, clinical outcomes vary considerably and are influenced by patient characteristics, such as age, sex, osteotomy gap size, and duration of preoperative sports cessation [4,5]. Therefore, sports-oriented rehabilitation should be individualized and designed to support patients' motivation even after hospital discharge.

Running is a high-impact activity because knee joint reaction forces can reach up to 10 times body weight during stance [6]. Safe running requires adequate quadriceps and hamstring strength and neuromuscular control. The quadriceps muscles contribute to controlled knee flexion during heel strike and loading response, thereby reducing tibiofemoral stress [7]. However, quadriceps strength typically decreases after MOWHTO, and it may require >6 months to recover [8,9]. Inadequate quadriceps strength during the period before bone union (approximately three months) makes it difficult to regain running ability early. Therefore, rehabilitation after MOWHTO aims to prevent quadriceps atrophy, maintain daily step count until bone union, and gradually reintroduce jogging and running after bone union. However, owing to insurance limitations, outpatient rehabilitation often concludes on postoperative day (POD) 150. Additionally, patients living far from specialized facilities may not have access to sports-oriented rehabilitation services. Patients with limited self-management may struggle to maintain physical activity after discharge, leading to suboptimal recovery. mymobility® is a smartphone application that offers exercise videos, step-count monitoring, education modules, and two-way communication. These features enable continuous remote support after discharge.

In this report, we present the case of an older woman who stopped running for two years due to knee pain and demonstrated quadriceps weakness and low activity before MOWHTO. mymobility® was used after MOWHTO to guide her return to jogging and running remotely because she could not attend postoperative rehabilitation at our institution. This case report demonstrates the feasibility and safety of the mymobility® platform, highlighting its potential utility in optimizing sports-oriented rehabilitation and facilitating a successful return to sports after MOWHTO.

## Case presentation

Patient information

**Figure 1:**
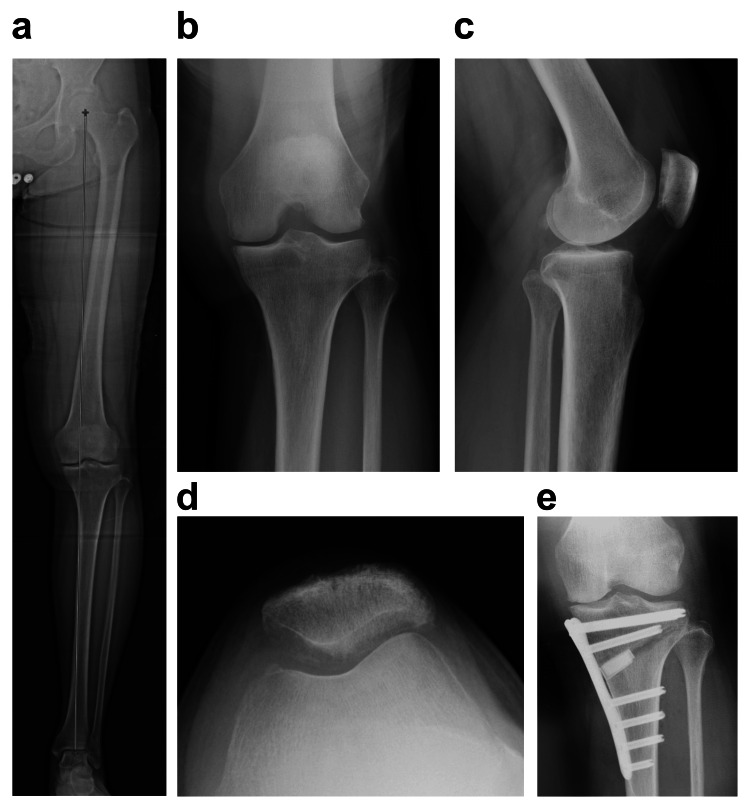
Radiographic data (a) The hip-knee-ankle angle was 5° varus. (b) The medial knee joint Kellgren-Lawrence grade was 3. (c) The tibial posterior slope was 6.4°. (d) The patellofemoral knee osteoarthritis grade was 1. (e) By expanding the tibia 9.5 mm, the hip-knee-ankle angle was corrected to 3.7° valgus.

A 71-year-old woman with a body mass index of 23.5 kg/m^2^ presented with medial knee pain during walking and stair climbing (Visual Analog Scale score, 80 mm). She used to run 7 km daily but stopped running for two years owing to knee pain. Radiographs showed Kellgren-Lawrence grade 3 medial KOA with a hip-knee-ankle angle of -5° varus (Figure 1). There was no OA in the contralateral knee, and the range of motion and quadriceps strength were sufficient, so there was no interference with daily life. The preoperative Knee Injury and Osteoarthritis Outcome Score (KOOS) scores were 53 for Symptoms, 50 for Pain, 61 for Activities of Daily Living (ADLs), 25 for Sports, and 6.25 for Quality of Life (QOL) [10]. The Oxford Knee Score (OKS) was 31 [10]. The Pain Catastrophizing Scale score was 8 [10]. Her median daily step count was 1776 steps (mymobility®) [10]. The number of steps was assessed using the median value of the values ​​measured over a seven-day period.

Surgical procedure

MOWHTO is indicated for active patients with medial KOA or osteonecrosis who meet the following criteria: knee extension ≥-5°, flexion ≥130°, no marked degeneration of lateral or patellofemoral compartments, and femorotibial angle <182°. The target alignment was planned with a weight-bearing line ratio of 65-70%. MOWHTO was performed as described by Staubli and Jacob [11] using an ASPIC® plate and synthetic bone graft (AviOS, HOYA Technosurgical Corporation, Shinjuku, Japan). Partial weight-bearing was initiated on POD 7, and full weight-bearing was initiated on POD 28.

Postoperative rehabilitation

Rehabilitation was initiated on POD 1 with range-of-motion and quadriceps isometric exercises. Preoperatively taught mymobility® exercises were continued at home. Weight-bearing progressed as follows: half weight-bearing on POD 14, ambulation with crutches on POD 21, walking with a cane on POD 28, and independent walking by POD 35. A rigid knee brace (X2K, SHILAC, Osaka, Japan) was worn, half weight-bearing exercises were performed after surgery, and the brace was removed after bone union was confirmed three months after surgery. Squats (0-90°) were started on POD 42. The patient was discharged on POD 42 and thereafter attended weekly outpatient therapy at a local hospital.

Remote rehabilitation and activity progression

**Figure 2:**
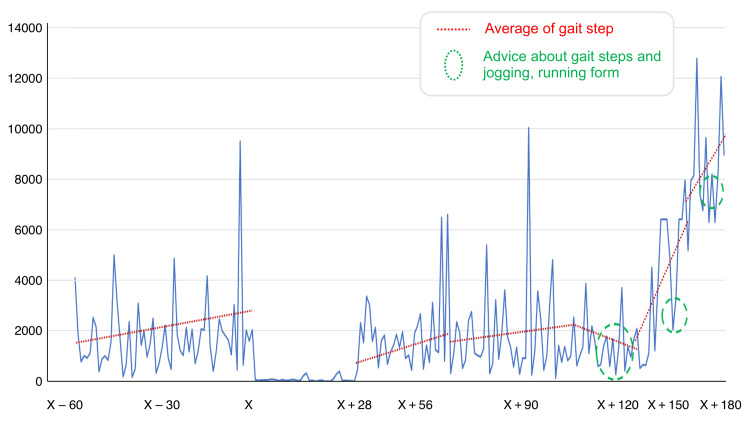
Longitudinal changes in gait step The green dotted circles indicate the dates of remote rehabilitation sessions we conducted with the patient using the mymobility® chat feature, and you can see that the number of steps increased after those sessions.

**Figure 3:**
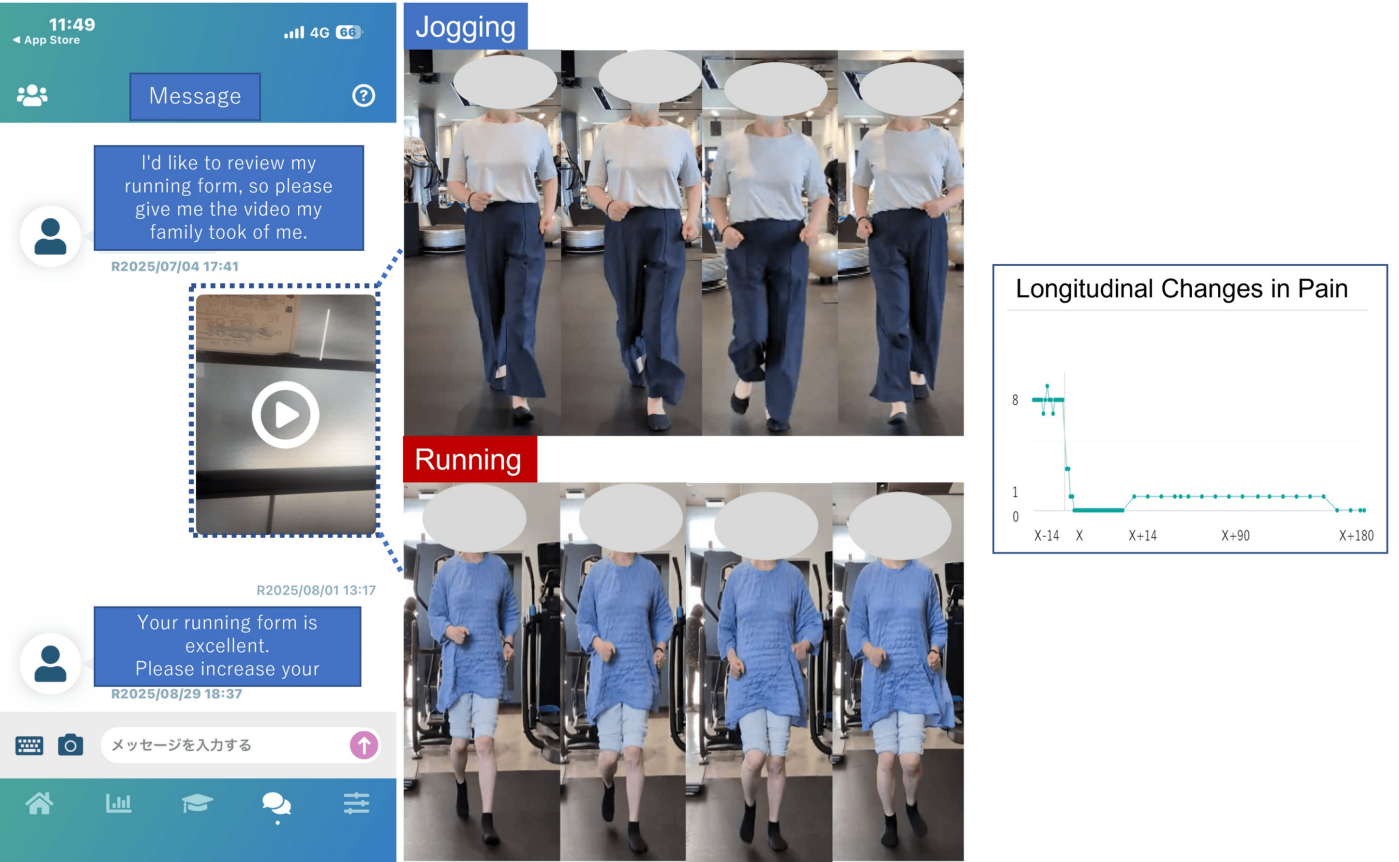
How to use mymobility® During remote rehabilitation using the mymobility® chat function, the patient sent videos of themselves jogging and running. It was clear that the knee joint remained stable in a valgus position during both running and jogging. It also showed that knee pain did not increase over time. The figure was created by the authors using CorelDRAW (Corel Corporation, Ottawa, Canada).

Despite bone union, the patient's step count gradually increased but remained low (approximately 2000 steps/day) until POD 90 (Figure 2). mymobility® was used to address her anxiety, establish a goal of 5000 steps/day, and instruct her to self-assess her quadriceps using a 40 cm single-leg stand test. Jogging attempts were recorded, and videos were uploaded for analysis. After 10 days of daily jogging trials without an increase in pain, she progressed to running. Jogging and running were achieved on PODs 150 and 180, respectively (Figure 3). The POD 180 KOOS scores were 82 for Symptoms, 90 for Pain, 90 for ADLs, 70 for Sports, and 50 for QOL [10]. The OKS was 44 [10]. In remote rehabilitation, patient guidance was provided using the chat function within the mymobility® smartphone application. The chat function also allows for the sending of videos and images to each other, and movement assessments were conducted by patients sending videos to medical professionals. It is important to note that medical fees are not calculated for this remote rehabilitation and patient guidance and movement assessments were conducted free of charge.

## Discussion

Herein, we present the case of a 71-year-old woman who had been unable to run for two years owing to knee pain. The mymobility® smartphone application was used to guide her return to jogging and running remotely after MOWHTO, and she successfully regained jogging ability on POD 150 and running ability on POD 180, with a high level of patient satisfaction. This case indicates the usefulness of the mymobility® smartphone application as a tool to support sports-oriented rehabilitation after MOWHTO.

The KOOS has been reported to be a useful tool for evaluating clinical outcomes in patients undergoing MOWHTO who participate in sports. Jacquet et al. reported that the minimal clinically significant differences in KOOS scores at two years after MOWHTO were 15.4 for Pain, 15.1 for Symptoms, 17 for ADLs, 11.2 for Sport and Recreation Function, and 16.5 for QOL [12]. In our case, the patient's KOOS scores improved beyond the minimal clinically significant difference in all domains (Pain, +25; Symptoms, +40; ADLs, +29; Sport and Recreation Function, +25; QOL, +43.8) even within the postoperative period (six months). These results confirm that the patient achieved meaningful early improvement in clinical outcomes. Patients with preoperative Tegner Activity Scale (TAS) scores <6 have been reported to have RTS rates of approximately 90%. However, those involved in high-impact activities (TAS score ≥6) have significantly lower RTS rates of 25-40% [13]. Our patient had a long-standing habit of running 7 km daily (TAS score of 6-7) but stopped running for two years. The duration of preoperative sports cessation has been reported to negatively influence RTS [14]. Older age and prolonged preoperative inactivity posed challenges to successful RTS. However, frequent guidance using mymobility® contributed to improved motivation and physical function, ultimately facilitating RTS.

Age is an important factor influencing MOWHTO outcomes. In previous studies conducted in Japan, the mean age of patients undergoing MOWHTO was commonly in the 70s to 80s, whereas international reports often included younger patients in their 40s [1,2,5]. Similar to our case, Kamada et al. reported that the average RTS time in older patients was approximately 14 months, with a return rate of 25% [15]. Nakayama et al. reported that 79.4% of a younger Japanese cohort in their 50s returned to high-impact activities within eight months [16]. However, no Japanese studies have specifically examined return to high-impact activities (TAS score ≥6) in older individuals. The available evidence indicates that older patients would take much longer to return to running. Therefore, our case is valuable because it documents early RTS in an older runner. This patient returned to jogging (TAS score of 5) at five months and resumed daily running (TAS score of 6-7) by eight months, which is relatively early compared with existing reports. These findings indicate that remote rehabilitation using mymobility® can shorten RTS time. However, caution should be exercised when interpreting the results as the patient received weekly outpatient physiotherapy at another hospital. Therefore, the observed effects cannot be attributed to mymobility® alone.

Regarding the specific components of remote rehabilitation through mymobility®, two aspects were particularly beneficial: (1) promoting increased daily activity and (2) using videos to assess running motion. Although the patient achieved stable bone union and had minimal pain during walking on POD 90, her daily step count remained low (approximately 2000 steps). Without postoperative remote rehabilitation, continued low activity might have delayed muscle strength recovery and prolonged her inability to resume running. Digital monitoring using mymobility® has been reported to increase step counts in patients undergoing knee or hip arthroplasty and MOWHTO [17,18]. This finding is consistent with our report, which showed an increase in step counts. The reason why the number of steps could not be increased was that the patient feared that increasing the step count could cause pain flare-ups or hinge fractures. The medical team addressed these concerns in real time through mymobility® communication, enabling safe activity progression. Short outpatient consultation time and the patient's reluctance to report minor concerns owing to social factors can limit communication between patients and clinicians. mymobility® enables patients to consult the surgeon, physiotherapists, and nurses, allowing the clinical team to identify and address issues that patients cannot clearly articulate. In situations like ours, where the patient received outpatient rehabilitation at a nonspecialized facility, remote monitoring and guidance on the progression of sports-specific rehabilitation are important. Furthermore, the ability to upload videos for running form assessment allowed clinicians to provide timely feedback, which contributed to the patient's psychological reassurance and successful RTS. Smartphone-based movement analysis has been reported to provide sufficient accuracy for rehabilitation [19]. Additionally, in patients whose pain increases during activity progression, correlating step-count data with pain levels through mymobility® can help adjust their activity levels appropriately [10]. Remote rehabilitation using mymobility® allows for simultaneous pain management and movement assessment, which is thought to be effective in helping patients return to sports activities.

A limitation of this study is that it is a case report of a single patient. Because the subject also underwent outpatient rehabilitation, it cannot be concluded that the effect of mymobility® alone was a factor. In the future, we believe it is necessary to increase the sample size and conduct observational studies. Furthermore, we would like to verify the significance of an increase in step count in a state where mechanical stress has been reduced after surgery by simultaneously measuring not only the increase in step count but also kinetic and mechanical data during walking (knee adduction moment and varus thrust) related to clinical outcomes [20].

## Conclusions

This case indicates that mymobility® can facilitate safe and timely return to sports after MOWHTO by improving motivation, providing remote supervision, and enabling individualized activity progression. mymobility® may be particularly effective for older patients or those who cannot access specialized postoperative rehabilitation services. However, it should be noted that this report is a single-patient design and is a case where outpatient physical therapy was administered simultaneously, so the conclusion is that the effectiveness of mymobility® is merely an additional treatment.
